# New Antimicrobial Materials Based on Plasticized Polyvinyl Chloride for Urinary Catheters: Preparation and Testing

**DOI:** 10.3390/polym16213028

**Published:** 2024-10-29

**Authors:** Iuliana Mihaela Deleanu, Elena Grosu, Anton Ficai, Lia Mara Ditu, Ludmila Motelica, Ovidiu-Cristian Oprea, Gratiela Gradisteanu Pircalabioru, Maria Sonmez, Cristina Busuioc, Robert Ciocoiu, Vasile Iulian Antoniac

**Affiliations:** 1 Faculty of Chemical Engineering and Biotechnology, National University of Science and Technology POLITEHNICA Bucharest, 1-7 Gh. Polizu, 011061 Bucharest, Romania; iuliana.deleanu@upb.ro (I.M.D.); ludmila.motelica@upb.ro (L.M.); ovidiu.oprea@upb.ro (O.-C.O.); cristina.busuioc@upb.ro (C.B.); 2 Faculty of Material Science and Engineering, National University of Science and Technology POLITEHNICA Bucharest, 313 Splaiul Independentei Street, 060042 Bucharest, Romania; elena_grosu@yahoo.com (E.G.); ciocoiurobert@gmail.com (R.C.); iulian.antoniac@upb.ro (V.I.A.); 3 National Center of Micro and Nanomaterials, National University of Science and Technology POLITEHNICA Bucharest, Splaiul Independentei 313, 060042 Bucharest, Romania; 4 Academy of Romanian Scientists, 3 Ilfov St., 050044 Bucharest, Romania; gratiela.gradisteanu@icub.unibuc.ro; 5 Faculty of Biology, University of Bucharest, 1-3 Intr. Portocalelor Street, 060101 Bucharest, Romania; lia_mara_d@yahoo.com; 6 Research Institute of the University of Bucharest, 90 Sos. Panduri, 050663 Bucharest, Romania; 7 National Research and Development Institute for Textile and Leather, Leather and Footwear Institute, 93 Ion Minulescu Street, 031215 Bucharest, Romania; maria.sonmez@icpi.ro

**Keywords:** urinary tract infections, silver nanoparticles, plasticizer, antimicrobial material, polyvinyl chloride, medical applications

## Abstract

Given the constant increased number of nosocomial infections in hospitals, especially associated with prolonged usage of inserted medical devices, our work aims to ameliorate clinical experience and promote faster healing of patients undergoing urinary catheterization by improving the properties of medical devices materials. Within this research, nine different composites were prepared based on polyvinyl chloride, using three different plasticizers (di-(2-ethylhexyl) phthalate, Proviplast 2646, and Proviplast 2755), and two different antimicrobial additives containing silver nanoparticles. The prepared materials were analyzed, and their physicochemical properties were determined: water absorption, relative density, plasticizer migration, hydrophobicity/hydrophilicity by contact angle measurement, Shore A hardness, tensile strength, and elongation at break. Structure and morphology were also investigated by means of FTIR, SEM, and EDX analyses, and thermal (TG-DSC) and biological properties were evaluated. The most important aspects of obtained results are showing that plasticizer migration was significantly reduced (to almost zero) and that the usage of antimicrobial additives improved the materials’ biocompatibility. Thus, based on the concluded favorable properties, the obtained materials can be further used for catheter development. Pressure–flow studies for different sizes and configurations are the next steps toward advanced in vivo and clinical trials.

## 1. Introduction

Among infectious diseases, urinary tract infections (UTIs) rank second in the world, affecting men and women equally [1]. Depending on the occurrence site, UTIs can be classified as urethritis (when urethra is affected), ureteritis (ureter inflammation), and cystitis and pyelonephritis (for bladder and kidney) [2]. Generally classified as uncomplicated or complicated and disregarding the infection site, UTIs can be caused by both Gram-negative and Gram-positive species, and some fungi or viruses. The most prevalent uropathogens include *Escherichia coli* (almost 40% of non-community infections), *Klebsiella pneumoniae*, *Proteus mirabilis*, *Enterococcus faecalis*, *Staphylococcus saprophyticus*, and other microorganisms [3,4]. Antibiotics, often applied empirically, based on local profiles and guidelines and not on culture testing, are used to treat UTIs. As a result of irresponsible or not well managed use of antibiotics, the antibiotic resistance in uropathogens has become one of the most significant public health issues. Despite the importance, so far there are limited data on antibiotic resistance of uropathogens for UTIs and their recurrences [5,6].

An overwhelming majority of these infections is represented by catheter-associated urinary tract infections (CAUTIs)—almost 80% [7]. These are in fact considered as complicated UTIs and are often associated with increased mortality and secondary bloodstream infections [3].

There are numerous cases in which patients with chronic conditions are immobilized in bed and require bladder catheterization with urinary catheter or Foley catheter, intermittent or indwelling. The procedure of bladder catheterization can be performed only in hospitals, mainly for therapeutic purposes (urinary retention, perioperative, urinary incontinence, chemotherapy drug delivery, etc.) or for diagnosis purposes (sample collection of urine and urodynamics analysis) [8,9]. Despite protocols and stringent guidelines implemented and followed by each clinic, the prevention of CAUTIs remains a challenge, having as a predisposing factor the immunosuppression state of patients who require catheterization.

There are different types of urinary catheters (UCs). Foley catheters, used for indwelling, are composed of two flexible tubes, or lumen, fabricated mostly from plasticized polyvinyl chloride (PVC), or from other flexible plastics like silicon rubber or coated latex. The principal lumen drains urine out of bladder. The distal end is atraumatic closed by forming in the melt and is perforated at a distance of 1 cm from the distal end, creating a bladder opening or an eyelet that drain urine. The round atraumatic distal end penetrate the urethra and enter the bladder. In the next proximity, the balloon connected to the second lumen is inflated to prevent slipping out. Encrustations that appear inside the lumen in the distal end can lead to the obstruction of the eyelet drainage [10,11].

The main issue associated with catheterizations result from microorganisms colonizing the surface of the device, forming the so-called biofilm. Biofilm formation is just the beginning in a series of complications that can occur. Initiated and sustained by the urine proteins presence, the biofilm becomes a microorganisms’ supply source, which determines urease production. Urease production subsequently determines urinary pH increase (urine alkalinization), and ammonia ions are further produced. The biological environment becomes liable for mineral crystallization, and the crystals that are formed adhere to the biofilm determining encrustations on the inner catheter surface. Flow blockage or decrease materials elasticity can result [11,12]. As it was observed that biofilms develop in less than 24 h after insertion, it is clear that solutions need to be found as soon as possible.

The occurrence of urinary infections and these encrustations depend on many factors, but especially on the type and composition of the polymeric material from which the UC is made; urine biological characteristics; flow rate and heat; as well as the characteristics of the presence of commensal bacteria [13,14]. There are numerous commercially available UCs, made of different materials, each with numerous advantages and with certain disadvantages [15,16]. In the case of plasticized PVC UCs, the plasticizer type plays an important role. The selection of type and quantity of plasticizers in compositions is very important to achieve the necessary flexibility and softness of the tubes. It is well known the use of phthalate plasticizers in PVC compounding, and also the disadvantages of the migration of these plasticizers in the biological environment during insertion period [17]. Di-(2-ethylhexyl) phthalate (DEHP) is considered a carcinogenic, mutagenic, or reprotoxic substance [18,19,20]. Thus, in last years, numerous studies have investigated the replacement of phthalate-type plasticizers, especially in medical devices, with new plasticizers, having similar structures and similar physicochemical properties [21]. Some involved branched and hyperbranched compounds [22,23,24,25], or vegetable oil-based plasticizers synthesized from renewable resources [26,27,28]. For instance, an alternative to PVC-based compositions was the use of a polyolefin-based elastomer material (POBE) in the manufacture of LoFric urinary catheters (Astra Tech, Sweden). Johansson et al., 2013 performed a study on volunteer patients that were catheterized with three types of UC, a PVC-based catheter, LoFric PVC-free, and LoFric PVC, to compare the tolerability of PVC-free materials. The results of the study exhibited low discomfort rates for all materials, disregarding PVC content, although the best results were observed for the PVC-free LoFric catheter [29]. Currently, the industry of manufacturing PVC plasticized granules develops several grades of materials for use in medical tubing devices. A good example is Modenplast Medical Srl, Italy, which produces a wide range of medical-grade PVC compounds for extrusion, including PVC compounds plasticized with DEHT (di(2-ethylhexyl) terephthalate) [30].

Numerous experimental investigations have aimed, on the other hand, for surface modification by different techniques to inhibit bacteria attachment and biofilm formation on the catheters [31,32]. Some of these approaches can be mentioned: hydrogel coatings [33,34,35], polytetrafluoroethylene coatings [34,36], polyethylene glycol coatings [37,38], and silver composite coatings [39,40].

One of the solutions is searching and development of reliable antimicrobial UCs. Two major directions have been pursued by researchers aiming for performant catheter development; they can even be used for long periods (indwelling): catheter material modifications and/or antimicrobial coating deposition on the actual material [11]. When antimicrobial additives are used, special attention is given to their short-term and long-term effects as well. Commercially available silver alloy-coated latex catheters (Bardex IC, Bard Medical, Crawley, UK), for instance, proved effective in short-term use, and although antibiotic coating performance proved superior, silver alloy UCs are still on the market due to low cost and patient preference (comfort) [15,31]. In the last years, silver nanoparticle (AgNP) incorporation in medical devices and the potential risks they could exert on patients have been extensively investigated. For example, David Roe et al., 2008 investigated the systemic toxicity of plastic catheters whose surface was functionalized with AgNPs. Their investigation proved sustained release of AgNPs (approximately 16% of the coating in 10 days). In vivo testing indicated silver excretion, and limited diffusion through tissues with very little risk of attaining toxic concentrations in organs [41]. Other recent studies performed on UCs containing AgNPs also revealed, besides anti-biofilm and antibacterial effects, very few quantities of silver ions released and non-toxicity [42,43]. Green synthesis of nanoparticles (like AgNPs) and of nanostructured composites are in fact the latest research approaches [44].

But no matter how effective the methods of modifying the surfaces of UCs and other tubular medical devices are, we must recognize that they can only be applied externally. In normal usage, however, tubular medical devices become infected both externally and internally. In the development of a fully antimicrobial UC, we also have to consider the tube thickness or Charrière size, which cannot deviate from international norms. And if we think about the shape of the UC, for example, the main tube is closed at the distal end, not allowing for the access of internal covering techniques. Last but not least, the manufacturing technology sued for tubular medical devices must be considered, as it is reflected in the cost price of the final product.

In our work, presented here, we started from the importance of manufacturing technology. Currently, the tubular component parts of catheter-type medical devices are manufactured by melt extrusion technology of compounded polymeric granules. The most used polymer is plasticized PVC. Extrusion is the defining technological stage in the manufacturing flow of catheters. The performance of catheters depends mostly on the properties of polymeric materials and the extrusion processing parameters. The companies producing tubular medical devices, such as catheters, continuously improve, through collaborative communication between extrusion engineers and clients (or beneficiaries or customers), the stages of transposing the tubes from design to production [45,46]. Although advanced technologies for the manufacture of tubing for catheters are currently available, such as extrusion, co-extrusion, multi-layering, and wire coating, we consider that common extrusion of the granules of plasticized *PVC compounded together with antimicrobial additives*, here based on AgNPs, is more practical and efficient for obtaining a tube with antimicrobial properties at any point of the material, both on the lumen and on the outer surface, and especially in the tube section where perforations are made to obtain the eyelet or bladder opening at the distal end. The technology of processing by extrusion allows for the realization of variable configurations in continuous processes, resulting in high-performance catheters at an acceptable price.

Two main goals were thus considered in conducting our research:

i. Inhibition of plasticizer migration using modern, high performance, biocompatible plasticizers, as an alternative to less safe DEHT, namely:
  - Proviplast 2646 citrate-based, a high performing plasticizer for medical applications according to its technical datasheet, listed in the *European Pharmacopoeia* [47]. It is a high-purity, safe alternative for DOP/DEHP in specialized medical applications, such as blood bags or tubes. Highly compatible with PVC, Proviplast^®^ 2646 has low heavy metal content and is recommended for applications in the healthcare area/medical/blood bags.
  - Proviplast 2755, considered a safe, bio-based, phthalate-free plasticizer by Proviron, exhibits high efficiency, thermal stability, and reduced carbon footprint. Proviplast^®^ 2755 meets the requirements for sensitive applications for PVC end-products [48].
ii. Development of a material with enhanced functionality, reduced plasticizer migration (close to zero), reduced encrustations formation ability within the lumen of tubes, biocompatible, to be used in the fabrication of the UCs, and containing antimicrobial additives based on silver nanoparticles (AgNPs) to provide antibacterial activity.

It must be emphasized that this investigation and its results follow previous intensive research on group members in finding a suitable polymeric matrix that can be safely and effectively applied in the medical filed [49,50,51]. To the best of our knowledge, the usage of Proviplast 2646 and Proviplast 2755, together with specific synthetized AgNPs in PVC compositions, as designed and tested in this work, has not been yet reported.

## 2. Materials and Methods

### 2.1. Materials

To prepare the polymeric materials to be implemented as UCs, the following chemicals were used (medical-grade): Polyvinyl chloride (PVC) from Merck Romania SRL (București, Romania), an affiliate of Merck KGaA, Darmstadt, Germany, average molecular weight Mw~62,000, average Mn~35,000, CAS Number: 9002-86-2. Plasticizers: di-(2-ethylhexyl) phthalate (DEHP) from Merck Romania SRL, an affiliate of Merck KGaA, Darmstadt, Germany (abbreviated as P1); Proviplast 2646, listed in the *European Pharmacopoeia* (abbreviated as P2) and Proviplast 2755 (abbreviated as P3) from Proviron Ostend, Oostende, Belgium; epoxidized soy bean oil (ESO) from Alfa Chemistry, Holbrook, New York, NY, USA; Ca/Zn stearate from Alfa Chemistry, Holbrook, NY, USA; and antioxidant Irganox 1076 from BASF, Ludwigshafen, Germany. Silver nanoparticles (AgNPs) embedded in ceramic matrix from Sanitized AG, Burgdorf, Switzerland (designated as AC) and silver nanoparticles (AgNPs) prepared in our laboratories (designated as AP) were used as antimicrobial agents.

### 2.2. Methods

#### 2.2.1. Sample Preparation

Silver nanoparticles (AP), used in these composites as antimicrobial additive, were prepared in our laboratories in accordance with a method previously described. Briefly: 0.02 g AgNO_3_ was dissolved in 100 mL distilled water, at 70 °C, under stirring. Sodium citrate, used as reduction agent, was then slowly added as solution (0.5 g in 20 mL). After 30 min, 5 mL of polyvinylpyrrolidone (PVP) solution (containing 0.1 g PVP) was added dropwise in the reducing solution. The final yellowish solution contains 100 ppm AgNPs, having an average diameter of 30 nm [52,53].

We performed nine compositions of PVC coded as C1 ÷ C9 according to Table 1. The substances were weighed with precision using a RADWAG balance (Radom, Poland).

The preparation of the composites was accomplished in two technological stages. First, all dry components were blended at room temperature, in a PVC bowl. The powder was mixed homogeneously with the required amount of plasticizer, after which the stabilizer and antioxidant were added. In the second stage, the dry blend was melt-compounded in the laboratory, within a vat with a capacity of 50 cm^3^ of a plastograph, a Brabender Plasti-corder kneader (Duisburg, Germany).

To obtain the control samples, the compositions were mixed for 10 min. For the compositions with antimicrobial additive, after a few minutes of mixing, when the mixture became completely gelled, the antimicrobial additive based on AgNPs was added, continuing the mixing in the melt, until a steady torque was achieved, i.e., until the duration of 10 min. The mixing parameters are presented in Table 2.

After the completion of mixing in the melt, the compositions were removed from the Brabender vat, and were pressed with the help of a Fontijne Grotnes press (Vlaardingen, The Netherlands) to obtain films with a thickness of 1 mm. The pressing parameters are presented in Table 3.

The films thus obtained were punched to obtain test specimens for physical–mechanical and biological analyses, as will be further described.

#### 2.2.2. Physical and Mechanical Analyses of Samples

***Water absorption*** was performed according to the ISO 62:2008 standard [54]. Water absorption represents the amount of water absorbed by a material under specific conditions. In the case of polymers, the factors that influence water absorption are the following: the type of polymer, the additives used, and the working temperature and the duration of exposure of the polymer in contact with water. The samples used from compositions C1 ÷ C9 had a circular shape, with a diameter of 10 mm and a thickness of 1 mm. The procedure consisted of drying the samples for one hour in an oven at 50 °C, and after cooling in the desiccator, the samples were weighed, and their mass values were recorded. Water absorption was determined by immersing the samples in distilled water in 50 mL Berzelius glasses, at room temperature 25 °C. The experiment lasted five days, after which the samples were removed from the distilled water, dried with filter paper and weighed. The masses of the samples were recorded. Water absorption was determined as a percentage increase in the mass of the samples, using Equation (1):

(1) $$Water\;absorption,\;\%=\frac{{m_{f}-m}_{i}}{m_{i}}\times 100$$

where $m_{i}$ represents the initial mass of the samples recorded in a dry state, before immersion in distilled water (g), and $m_{f}$ represents the mass of the samples recorded after each removal from the distilled water and gentle drying (g).

***Relative density*** was determined using an analytical balance with 4 decimals (Radwag AS 220/R2, Radom, Poland). The testing method was according to standard ISO 1183-1:2019 [55].

***Plasticizer migration***—The analyzes carried out to determine the migration of plasticizers from samples C1 ÷ C9 were carried out using the method set forth in standard ISO 177:2016 [56]. The test method highlights the tendency of plasticizers to migrate from the plastic material analyzed to other plastic materials or other nature when they are placed in intimate contact. The samples used were obtained by punching the plates obtained by pressing C1 ÷ C9 and had the shape of discs with a diameter of 50 mm ± 1 mm and a thickness of 1 mm. The samples were conditioned in a desiccator for 24 h according to ISO 291 [57], before the start of the test. After conditioning, samples C1 ÷ C9 were weighed three times each on the RADWAG analytical balance and the values were recorded with a tolerance of 0.001 g. Also, the thicknesses of the samples were measured three times at different points on the surface, with the help of a micrometer, and the average thickness was determined with a tolerance of 0.01 mm. To determine the loss of plasticizer, one sample of each material C1 ÷ C9 was inserted between two absorbent polystyrene discs capable of absorbing plasticizer. They were fixed with a device creating a sandwich type assembly. This assembly was in turn inserted between two glass plates on which a 5 kg weight was placed, then it was inserted into the Memmert oven (Memmert GmbH & Co. KG—Schwabach, Germany), and the temperature was adjusted to 70 °C ± 2 °C. After 24 h, the assemblies were removed from the oven. After being removed from the sandwich structure, the PVC samples were conditioned for 4 h in a desiccator. After conditioning, the samples and the polystyrene discs were weighed three times, and the obtained values were noted. The migration of the plasticizer was highlighted by the mass loss of the plasticized PVC samples, according to Equation (2):

(2) $$Mass\;loss,\;\%=\frac{{w_{1\;}-w}_{2}}{w_{1}}\times 100$$

where $w_{1}$ is the initial mass of sample (g), and $w_{2}$ is the mass of the sample after the test (g).

***The contact angle*** was determined using Owens, Wendt, Rabel, and Kaelbe (OWKR) method and Kruss Drop Shape Analyzer, DSA-1000 (A. Kruss Optronic GmbH, Hamburg, Germany) apparatus. The hydrophilic/hydrophobic character of samples was assessed. The contact angle measurements in triplicate were performed using water, diiodomethane, and ethylene glycol, at room temperature 25 ± 5 °C and humidity 45 ± 5%. The final result was a mean of measurements for each sample.

***The surface free energy*** (SFE) of samples was determined using the method by Owens, Wendt, Rabel, and Kaelbe (OWKR).

***The Shore A hardness*** test was carried out according to ISO 48-4:2018 [58], using a Zwick 7206 Hardness Tester (ZwickRoell GmbH & Co. KG, Ulm, Germany). Samples of 6 mm thickness were used for hardness measurements. Five measurements were made on each sample type.

The ***mechanical properties*** of the samples C1 ÷ C9 were determined according to the ISO 527—1 test method [59] by means of a universal testing machine (Model Instron 1011, Instron, Norwood, MA, USA) at a test speed/crosshead speed of 10 mm/min.

***Microstructural investigations*** of all materials (C1 ÷ C9) and a rough particle size evaluation of antimicrobial additives were performed by Scanning Electron Microscopy (SEM) and Energy-Dispersive X-ray Analysis (EDAX), using a FEI Quanta Inspect F Scanning Electron Microscope (FEI Company, Hillsboro, OR, USA).

***Attenuated Total Reflection Fourier-Infrared (ATR-FTIR) Spectroscopy***—The spectral analyses of materials were performed using a Nicolet iS50 FTIR spectrometer (Thermo Fisher Scientific Inc., Waltham, MA, USA). Also, a Nicolet iS50R FTIR microscope (Thermo Fisher Scientific Inc., Waltham, MA, USA) was used to record the 2D maps.

An STA 449C F3 apparatus from Netzsch (Selb, Germany) was used for ***thermal analysis thermogravimetry*** (TG) and ***differential scanning calorimetry*** (DSC).

#### 2.2.3. Biological Analyses

The tests and values necessary for the assessment of biocompatibility consist of physicochemical analyses and biological tests, according to SR EN ISO 10993-1:2004 (cytotoxicity, biofilm development, antimicrobial activity, and biocompatibility of materials) [60].

***The antimicrobial assays*** were performed using standard microbial strains from the Microbial collection of the University of Bucharest, Faculty of Biology, Microbiology Department: *Staphylococcus aureus* ATCC 25923, *Escherichia coli* ATCC 25922, and *Candida albicans* ATCC 10231. All chemicals and reagents needed, having analytical grade, were supplied by Merck Romania SRL, an affiliate of Merck KGaA, Darmstadt, Germany.

For experiment assay, two successive passages on nutritious agar medium were performed, followed by incubation for 24 h, at 37 °C.

Quantitative assessment of the capacity of the selected strains to adhere and to generate mature biofilm on the surface of the tested polymeric samples was performed using the viable cell count (VCC) method. Overnight microbial cultures were diluted in fresh nutrient broth media at 1.5 × 10^5^ CFU/mL final density and 2 mL of the obtained suspension were seeded in 24 multi-well plates containing the treated materials, previously sterilized by UV irradiation (exposure time 30 min/each part). The plates were incubated at 37 °C, 24 h (for adherence evaluation), and 72 h (for biofilm evaluation). Subsequently, the polymeric samples were gently washed with sterile phosphate-buffered saline (PBS) in order to remove the non-adherent bacteria and placed in 14 mL centrifuge tubes containing 1 mL of sterile PBS. The samples were vigorously mixed by vortexing for 1 min and sonicated for 10 s. Serial dilutions obtained from each sample were inoculated on Muller Hinton agar and Sabouraud agar plates in triplicates, and viable cell counts (VCCs) were assessed after incubation at 37 °C for 24 h. Each test was performed in triplicate and repeated on at least three separate occasions.

Biological results were analyzed using a one-way ANOVA repeated measures test. All statistical analyses were performed using GraphPad Prism Software, v. 5.03 428 (GraphPad Software, La Jolla, CA, USA, www.graphpad.com, accessed on 20 May 2020). *p*-values were determined via two-way ANOVA and Dunnett’s multiple comparisons tests. Significance difference was noted as * for *p* < 0.05, and ** for *p* < 0.01.

***Cytotoxicity analyses determined by test dead/life***—the biocompatibility of the materials was evaluated on HDF human fibroblasts. These cells were cultured in DMEM (Dulbecco’s Modified Eagle Medium, Gibco) supplemented with 10% fetal bovine serum (Gibco cell culture products, part of Thermo Fisher Scientific Oy, Vantaa, Finland) and 1% Pen/Strep (penicillin/streptomycin solution, 50 µg/mL—Sigma-Aldrich, St. Louis, MO, USA) at a temperature of 37 °C, with a humidity of 95% and 5% CO_2_. After cultivation, cells were washed with saline, trypsinized (trypsin-EDTA 0.25%, Thermo Scientific, Boston, MA, USA), and counted using Trypan Blue and a hemocytometer. The biomaterials were co-cultured with the cells at a seeding density of 5 × 10^5^ cells/disc under the same environmental and temperature conditions as previously mentioned. All chemical and reagents needed were supplied by Merck Romania SRL, an affiliate of Merck KGaA, Darmstadt, Germany.

Biocompatibility evaluation was performed after 2 days of cultivation using MTT, LDH, and Live/Dead assays. The MTT assay was used to assess cell viability and proliferation in the presence of the materials. This test is based on the ability of the compound MTT (3-(4,5-dimethylthiazol-2-yl)-2,5-diphenyltetrazolium bromide) to be metabolized by living cells, forming soluble purple formazan crystals. The resulting absorbance was measured at a wavelength of 550 nm using a NanoQuant Infinite M200 Pro instrument (Tecan Group Ltd., Männedorf, Switzerland). To evaluate the cytotoxicity of the materials, the LDH Cytotoxicity Detection Kit (Roche, Basel, Switzerland) was used. LDH activity was measured in the culture supernatant at a wavelength of 490 nm, with a wavelength reference of 600 nm, using the same instrument previously mentioned.

When applicable, the tests were performed in triplicates and the results were represented as a mean ± standard deviation. Data analysis was conducted by one-way analysis of variance (ANOVA) and the significance of each mean value was defined (*p* < 0.05) with Duncan’s multiple-range post-hoc test applying SPSS software (version 280 21.0, IBM; Armonk, NY, USA).

## 3. Results and Discussion

### 3.1. Physical Properties Evaluation

#### 3.1.1. Visual and Microscopic Examination

**Figure 1:**
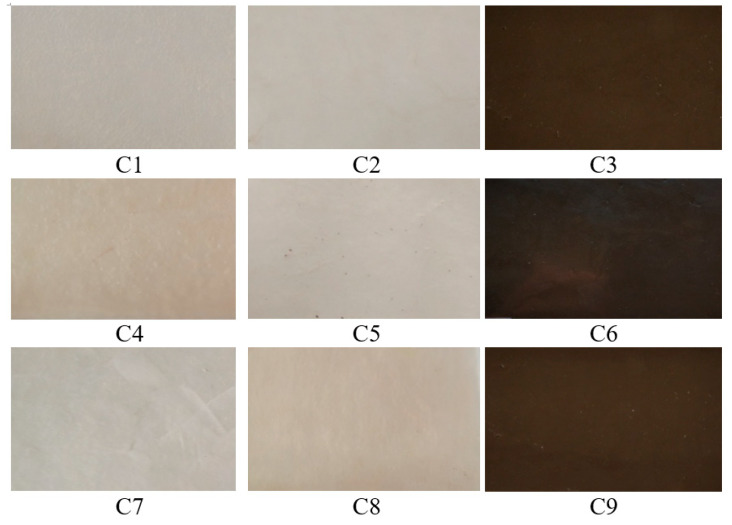
Visual aspect of experimental C1 ÷ C9 samples (images obtained by a digital photo camera. Not to scale).

Figure 1 shows photos of samples C1 ÷ C9, with dimensions 20 mm × 30 mm × 1 mm. As can be seen from the visual examination, the samples have a glossy, homogeneous appearance; the surfaces are smooth without deformations, free of impurities, without inclusions or cracks, and without air bubbles. The colors of samples C1, C4, and C7 are natural, opaque, and without traces of material flow in the melt. The colors of samples C2, C5, and C8 are natural, opaque, and homogeneous, without highlighting the areas of separation between phases. The AC additive encapsulated in the ceramic matrix is not visually evident. The colors of samples C3, C6, and C9 are almost opaque black due to the content of AP powder additive. There are no phase separations or traces of flow in the material.

#### 3.1.2. Contact Angle

PVC and plasticized PVC are mostly hydrophobic materials, which is an impediment when exposed to biological systems, promoting bacterial adhesion and biofilm formation [61]. There are studies (older and more recent) conducted on patients confirming that hydrophilic materials are preferred versus those non-hydrophilic, especially in intermittent or self-catheterization. Hydrophilicity allows for the surface to become smooth and slippery, so these catheters proved more comfortable than the standard ones: insertion is easier, and no other lubrication is needed. Thus, the urethral trauma is reduced, and device sterility can be maintained more easily. A lower rate of UTI was also reported in the case of hydrophilic catheters, although the association was not significant [62,63,64,65,66]. Other emerging studies are demonstrating the direct correlation between hydrophobicity and anti-fouling/antimicrobial properties. In other words, increased hydrophilicity was found to inhibit microbial adherence [31,67]. However, the findings on this issue are far from conclusive. Interdisciplinary efforts and combined engagement are further needed to investigate biofilm-related complications in catheterization (intermittent and/or indwelling) [68].

**Figure 2:**
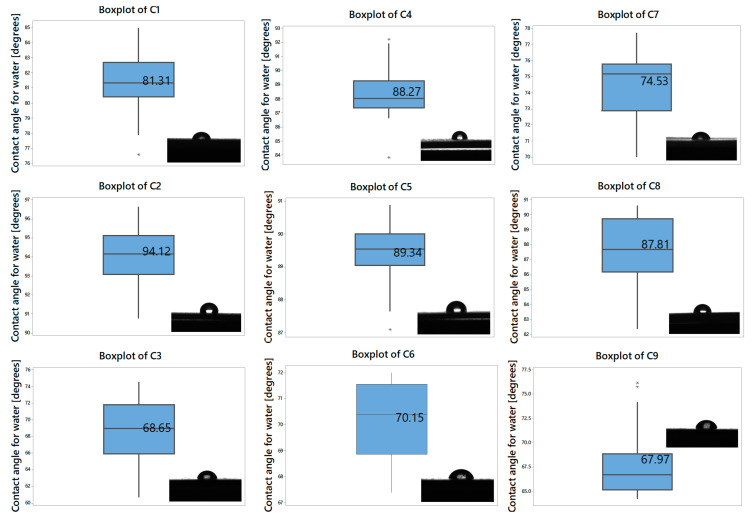
Contact angle values and microscopic images of water drop on experimental samples. Stars in the boxplots indicate the statistical significance (* for *p* < 0.05, ** for *p* < 0.001).

Figure 2 exhibits the boxplot of contact angle values obtained for our composites, and the images photographed during analysis performance. The values indicate, with the exception of C2 (with a contact angle of over 94°), the hydrophilic nature of the tested composites [69,70].

The results show the influence of the type of plasticizer and the antimicrobial additive used in the composition on hydrophilicity. Thus, for the compositions plasticized with P1, the hydrophilic character of the C1 material can be observed. The addition of the AC additive to C1 and obtaining the C2 material led to an increase in the contact angle and implicitly to obtaining a hydrophobic character. This is due to the presence of the encapsulating ceramic material of the AC additive. In contrast, composition C3, in which the AP additive powder was homogeneously mixed with composition C1, clearly shows a material with an increased hydrophilic character compared to the control sample.

In the case of the C4 ÷ C6 compositions plasticized with the P2 plasticizer, the same behavior was maintained, namely, the control sample C4 and the C5 sample presented a hydrophilic character close to the limit, but the C6 composition had an obviously improved hydrophilic character.

In the last case of the compositions C7 ÷ C9, the plasticizer P3 conferred improved hydrophilic character compared to the previous compositions. Control sample C7 and sample C9 presented smaller contact angles compared to the analogous samples plasticized with P1 and P2. Instead, sample C8 had a lower hydrophilicity, with a contact angle close to the upper limit.

From the analyzed results, it can be concluded that the three types of plasticizers had a good influence in the PVC-based compositions, and the wettability of the surfaces was consistently influenced by the type of antimicrobial additive.

#### 3.1.3. Surface Free Energy

The surface free energy for the samples C1 ÷ C9 was calculated using the OWKR method [71]. The determinations were made according to the OWKR method, using the three indicated liquids. The average value and the standard deviation were determined for five measurements.

**Figure 3:**
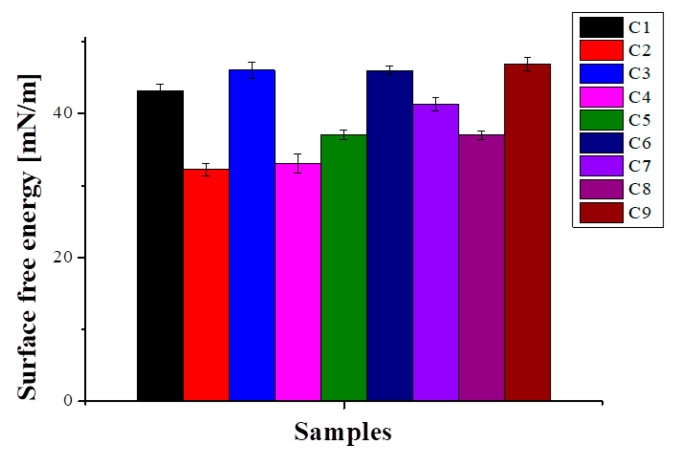
Graphical representation of surface free energy values.

The values of surface free energy for each sample are presented in Figure 3.

Recent studies emphasizing that hydrophilic biomaterials are more resistant to biological adhesion indicate that surface free energy value (as another way of characterizing hydrophobicity/hydrophilicity) is a good indication of an anti-adhesive material. Disregarding biological environmental conditions and the bacteria itself (biological factors), it was found that infection and bacterial colonization are negatively correlated with surface free energy [72]. Furthermore, values between 23 and 30 mN/m were associated with the lowest bacterial adhesion [73]. In our case, the obtained values are in the same range or higher (especially for C3, C6, and C9)—which could indicate the materials’ low fouling properties—by reduction in electrostatic attractions between them and bacteria at close contact [74].

#### 3.1.4. Shore A Hardness

**Figure 4:**
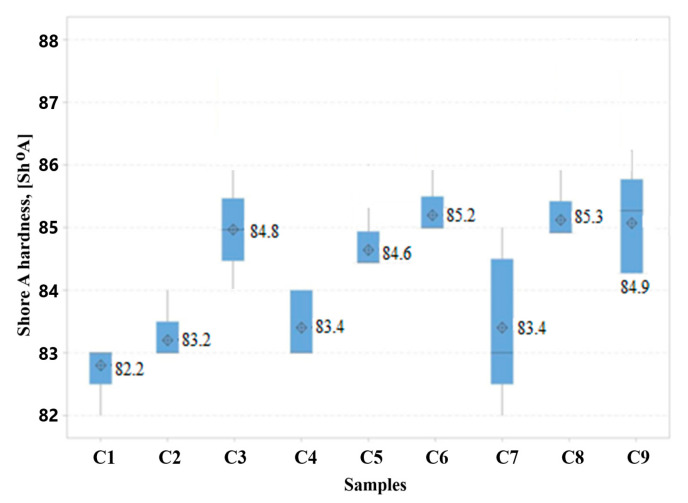
Boxplot showing Shore A hardness test results.

Shore A hardness and flexibility are very important properties for catheters, as they indicate the possibility of using catheters for the administration of drugs, nutrition, the elimination of biological fluids, or the administration of parenteral nutrition, in non-traumatic and comfortable conditions for the patient. The Shore A hardness value is inversely proportional to the plasticizer content [75]. Also, if the molecular mass of the plasticizer is small, it penetrates more easily into the free volume between the PVC chains and decreases the hardness of the composition [76,77]. According to the *European Pharmacopoeia*, Shore A hardness for urinary catheters must have a value 75–87 Sh⁰A [78]. Figure 4 shows that the Shore A hardness values for the compositions plasticized with P1 are lower than those of the compositions plasticized with P2 and P3 plasticizers. In any case, all samples present acceptable values, as recommended by regulation.

#### 3.1.5. Relative Density, Water Absorption, and Plasticizer Migration

##### Relative Density

All compositions C1 ÷ C9 have the same plasticizer content, namely, 30% wt. In Table 4, the relative density values for the test sample compositions C1, C4, and C7 correspond to common compositions of plasticized PVC. In each group of compositions plasticized with the same plasticizer, the relative densities increase compared to the control test due to the presence of antimicrobial additives AC and AP, which acted as filler in the composition and led to an increase in volume [79]. The compositions C1 ÷ C3 plasticized with P1 presented lower relative density values. Plasticizers P2 and P3 belong to the class of azobenzene and have a high polar character due to the aromatic ring in the molecule. The polar zones in the C4 ÷ C9 compositions that appear due to the P2 and P3 plasticizers determine the formation of secondary bonds with the PVC compositions and the increase in compatibility [77].

Water absorption in all samples exhibits very small values (as can be seen in Table 4), which complies with *European Pharmacopoeia 11* from 2023 [78].

Plasticizer leaching from the polymeric matrix is characterized by low values in case of P1 and very low migration values in the other cases (P2 and P3) a small influence can also be attributed to antibacterial agent, but overall he values are acceptable for medical devices, including high-exposure clinical procedures [17,80].

### 3.2. Mechanical Properties

#### Tensile Strength, Elongation at Break, and Residual Elongation

Some of the physicomechanical properties of plastic materials are crucial for their application. Among these, as mentioned above, Shore A hardness is a defining factor in the selection of plasticized PVC compositions to be used in the manufacture of medical tubing devices. At the same time, the analysis of mechanical properties such as tensile strength, elongation at break, and residual elongation provides information about the behavior of materials during use in contact with the human body. We can state that mechanical properties of the studied materials are the most important for tubular medical devices in order to perform the medical act in comfortable conditions for the patient and to avoid various traumas when handling these devices. The mechanical properties of plasticized polymer compositions are influenced by the chemical structure and molecular weight of the components and the branching or cross-linking of the chains [81].

**Figure 5:**
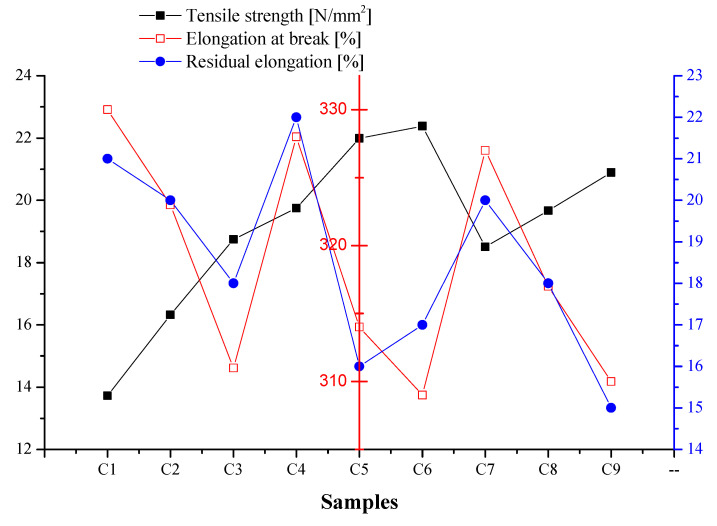
Values of mechanical properties determined for experimental samples (left, black axis shows tensile strength, N/mm^2^; middle, red axis shows elongation at break, %; and right, blue axis shows residual elongation, %).

Figure 5 presents the experimental results of the mechanical properties obtained for samples C1 ÷ C9. As can be seen, the compositions of PVC plasticized with P1 present the lowest mechanical property values, compared to the compositions plasticized with P2 and P3 plasticizers. For C2 ÷ C3 compositions, comparing with C1, the tensile strength increases by 16% for C2 and 27% for C3, respectively; the elongation at break decreases by 2% for C2 and 6% for C3; and the residual elongation decreases with approx. 5% for C2 and 14% for C3, all due to the antimicrobial additive content. Sample C3 exhibited small decrease in flexibility in comparison with sample control C1, as the antimicrobial additive AP had a higher dispersing capacity in the bulk polymeric composition. The same trend for tensile strength, elongation at break, and residual elongation was observed for C4 ÷ C6 and C7 ÷ C9 compositions as well. Overall, the highest values of tensile strength and the corresponding values for elongation at break and residual elongation were recorded for compositions C4 ÷ C6 added with P2 plasticizer. In descending order, the compositions C7 ÷ C9 and C1 ÷ C3 followed. Although all the compositions were constructed similarly, the variations in the values of the mechanical properties can be assigned to the physical properties of the plasticizers and the type of antimicrobial additive used. Since these additives are not chemically linked to the PVC chain, they only act as fillers, which results in a slight (acceptable) increase in the stiffness of the materials.

From the obtained results, it can be seen that none of the plasticizers or antimicrobial additives had a negative influence on the mechanical properties, but when designing new polymer compositions, their character must be carefully evaluated. Previous reported studies carried out on plasticized PVC urinary catheters covered with an antimicrobial layer showed that the tensile strength of the control sample had a value of approximately 4.6 kPa, and the coated samples had a tensile strength of up to 5.16 kPa, much lower values than obtained in our study [82]. Since an ideal catheter material should possess high tensile strength, we can conclude that our composites could be promising for the intended use [83].

### 3.3. Microstructural Properties

#### 3.3.1. Scanning Electron Microscopy (SEM) and Energy-Dispersive X-Ray Analysis (EDAX)

**Figure 6:**
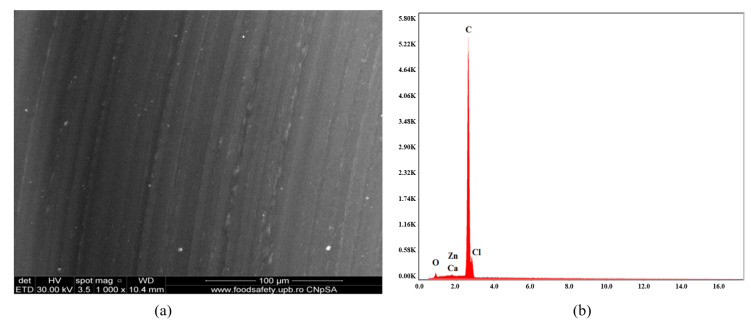
SEM micrograph at 1000× magnification (**a**) and EDX diagram of chemical composition (**b**) for sample C1.

**Figure 7:**
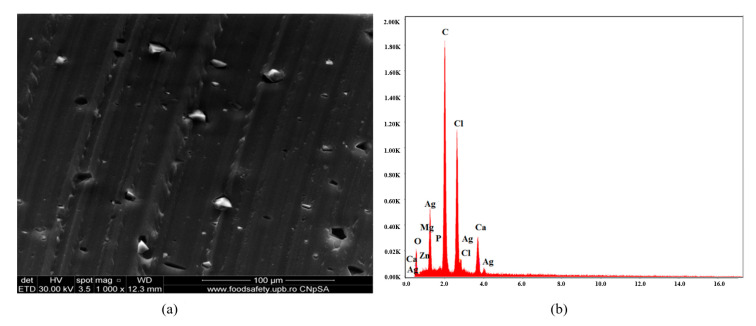
SEM micrograph at 1000× magnification (**a**) and EDX diagram of chemical composition (**b**) for sample C2.

**Figure 8:**
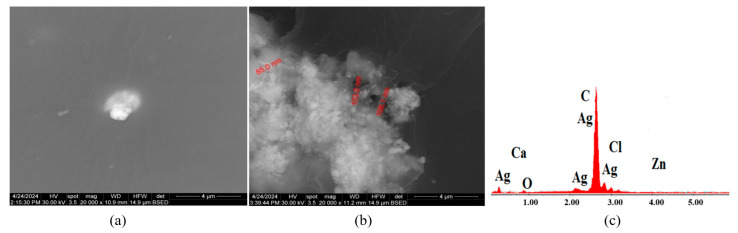
SEM micrographs of sample C3 with presenting localization of zone analyzed, magnification 20,000× (**a**), highlighting the AgNP dimensions at magnification 20,000× (**b**), and section of EDX diagram of chemical composition (**c**).

**Figure 9:**
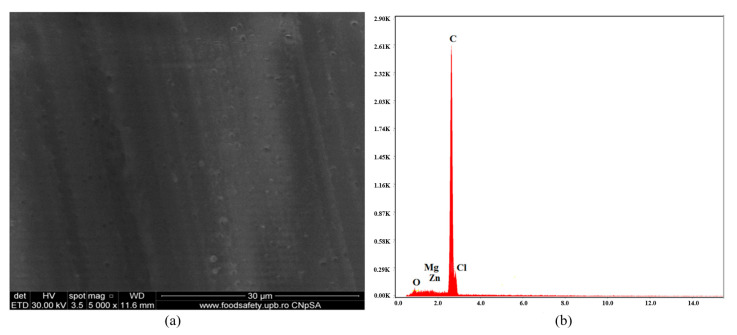
SEM micrograph at 5000× magnification (**a**) and EDX diagram of chemical composition (**b**) for sample C4.

**Figure 10:**
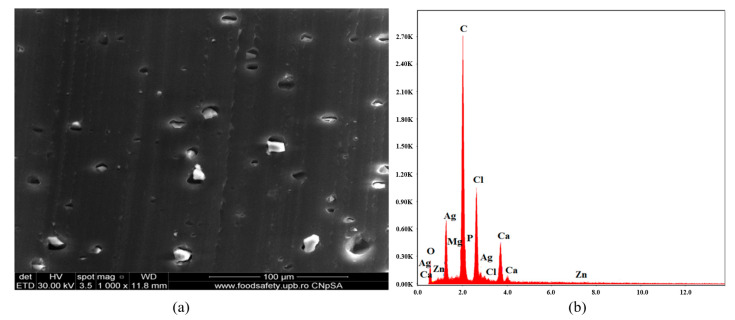
SEM micrograph at 1000× magnification (**a**) and EDX diagram of chemical composition (**b**) for sample C5.

**Figure 11:**
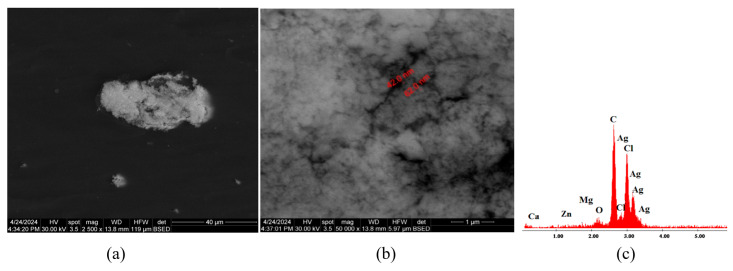
SEM micrographs of sample C6 at 2500× magnification (**a**), highlighting the AP dimensions at magnification 50,000× (**b**), and EDX diagram of chemical composition (**c**).

**Figure 12:**
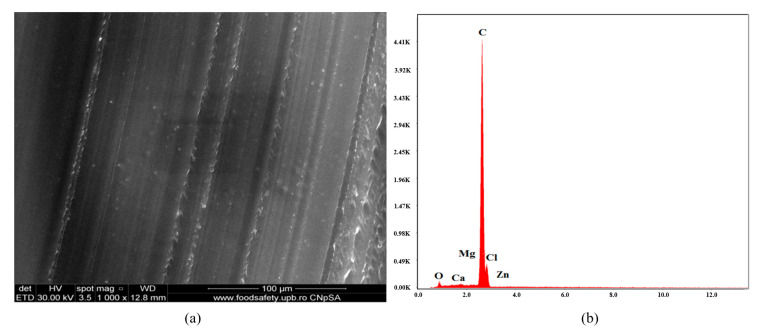
SEM micrograph at 1000× magnification (**a**) and EDX diagram of chemical composition (**b**) for sample C7.

**Figure 13:**
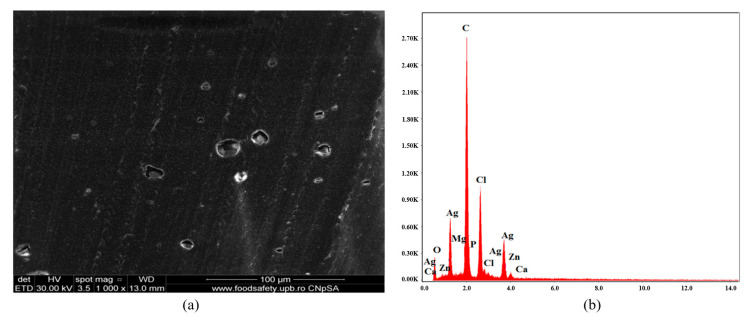
SEM micrograph at 1000× magnification (**a**) and EDX diagram of chemical composition (**b**) for sample C8.

**Figure 14:**
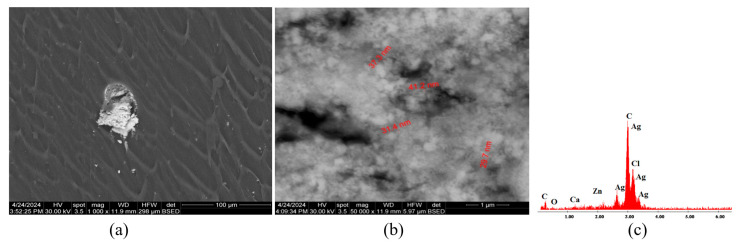
SEM micrographs of sample C9 with presenting localization of zone analyzed at magnification 1000× (**a**), highlighting the AP dimensions at magnification 50,000× (**b**), and EDX diagram of chemical composition (**c**).

Figure 6, Figure 7, Figure 8, Figure 9, Figure 10, Figure 11, Figure 12, Figure 13 and Figure 14 show the SEM micrographs and EDX diagrams of the plasticized PVC compositions. Figure 6a exhibit a homogeneous structure and good miscibility of plasticizer P1 with PVC. Microparticles of stabilizers (calcium/zinc stearate) uniformly distributed in the polymeric matrix can be observed. Figure 6b confirm the composition of sample C1. The EDX diagram presents all the chemical elements from PVC, plasticizer, stabilizers, and antioxidants: C, Cl, O, Ca, and Zn.

Figure 7a presents SEM micrograph of material C2 constituted from C1 with antimicrobial additive AC. The material is characterized by homogeneity, and good compatibility between components can be concluded. AC, located in the material, is uniformly distributed. Figure 7b shows an EDX diagram with an abundance of chemical elements: C, Cl (from PVC), C, O (from plasticizer), Ag from AgNPs, Ca, Mg, P, O (from ceramic material encapsulating AgNPs), Ca, and Zn (from stabilizer Ca/Zn stearate).

Figure 8a presents SEM micrographs of material C3 constituted from C1 with antimicrobial additive AP. The microstructure of sample C3 is uniform with no discontinuities or impurities, which indicates a homogenous material with uniformly distributed AP powder. In Figure 8b, AgNPs can be observed, with dimensions ranging from 80 nm to 110 nm. The EDX diagram from Figure 8c shows the chemical elements found in material C3: C, Cl, O, Ag, Ca, and Zn.

Figure 9a presents sample C4 as the test specimen. The material has the same composition as C1; instead of plasticizer P1, plasticizer P2 was used. The microstructure appears more homogeneous than C1, indicating better PVC and plasticizer miscibility, good plasticization effect, and more efficient mixing. The EDX diagram presented in Figure 9b shows its chemical composition: C, Cl, O, Ca, and Zn.

The same homogeneity also characterizes sample C5 (Figure 10a), having the same composition as C4 where AC was added. Moreover, uniform distribution of the additive in the continuous matrix can be observed. No discontinuities or irregularities can be seen. The EDX diagram presented in Figure 10b shows the same chemical elements as in C2: C, Cl, O, Ag, Ca, Zn, P, and Mg.

Figure 11a presents a SEM micrograph of the material C6 constituted from C4 with antimicrobial additive AP. The microstructure of material C6 is uniform, with no discontinuities or separation zones; the material presents as homogenous, and the additive seems uniformly distributed.

Figure 11b presents a high-magnification (50,000×) image of AP additive agglomerations with dimensions in the 40–60 nm range. The EDX diagram in Figure 11c indicates the chemical elements of C6: C, Cl, O, Ag, Mg, and Zn.

Figure 12a presents a SEM micrograph of material C7, considered the test specimen. The composition is similar to that of C1, but the plasticizer was replaced with P3. The microstructure is less homogeneous than C1 and C4. The EDX diagram in Figure 12b presents the chemical composition of the material: C, Cl, O, Ca, and Zn.

Figure 13a presents the microstructure of composite material C7. It can be noted that the AC additive is well distributed in the uniform polymeric matrix. The sample is free of discontinuities or irregularities. The EDX diagram in Figure 13b presents the same composition of chemical elements as C2 and C5, namely: C, Cl, O, Ag, Ca, Zn, P, and Mg.

Figure 14a presents a SEM micrograph of the material C9. The material is homogenous, and good mixing and miscibility can be observed between the PVC and plasticizer. In Figure 14b, magnified at 50,000×, AP additive can be spotted, with dimensions ranging around 30–40 nm. The EDX diagram presents the chemical composition of C9: C, Cl, O, Ag, Ca, and Zn. 

As a general conclusion, considering Figure 7a, Figure 10a and Figure 13a, it can be said that the additive AC can be seen distinctively in the PVC polymeric matrix. On the other hand, Figure 8a,b, Figure 11a,b and Figure 14a,b reveal the agglomeration tendency of the AP powder in PVC compositions. A similar behavior of the AP powder was previously reported when incorporated in polyvinylpyrrolidone (PVP)-based composites [53].

#### 3.3.2. Chemical Composition by ATR-FTIR Method

**Figure 15:**
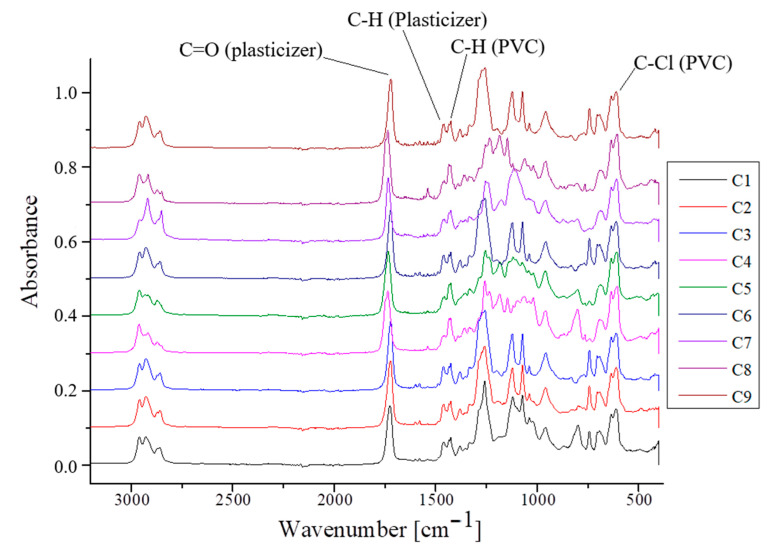
FTIR spectra for plasticized PVC samples. Characteristic absorption bands of PVC and plasticizer are emphasized, in accordance with Marcilla et al., 2008 [84].

Figure 15 displays the chemical interactions between components of the samples C1 ÷ C9, evaluated by specific bands from ATR-FTIR spectra.

The results considered between 3000 cm^−1^ and 450 cm^−1^ revealed a similar composition for all investigated samples, as the analyzed spectra did not indicate significant differences. Thus, characteristic peaks for the plasticized PVC can be identified, namely, molecular vibrations corresponding to functional groups of the PVC polymeric matrix: 715–556 cm^−1^ for C–Cl bonds and 1444–1414 cm^−1^ for C-H bonds [85,86,87,88,89,90]. For instance, in the case of samples C1, C2, and C3, containing DEHP-plasticized PVC, peaks of 2959 cm^−1^ corresponding to the asymmetric C–H stretching vibration of CH_3_ can be observed, as well as 2859 cm^−1^ for the C–H stretching vibration of CH_2_ [91]; 1725 cm^−1^, attributed to covalent bond C=O, belonging to the carbonyl group [92,93]; 1426 cm^−1^, an angular deformation attributed to –CH_2_Cl; 1379 cm^−1^ and 611 cm^−1^ to 635 cm^−1^ for C-Cl stretching [88]; 1259 cm^−1^, corresponding to hydrogen bond C-H angular deformation, located in functional group CHCl; and 1073 cm^−1^ to 1120 cm^−1^, attributed to C-C stretching vibration. In the case of C4 ÷ C6 compositions, the same peaks characteristics are maintained, and only small, unsignificant shifts in absorption bands can be seen at lower or higher values of wave numbers, due to P2 plasticizer usage. The same observation can be stated in the case of C7 ÷ C9 samples as well. Slight changes in peak characteristics, due to plasticized P3 usage, comparing to C1 ÷ C3 considered as control samples, can be seen. No significant morphological changes or modifications in FTIR spectra appear for C2, C3, C5, C6, C8, or C9 due to the presence of AgNPs additives. This reveals that no chemical bonds occur when the antimicrobial component is added to the PVC matrices; thus, the blending has only a physical characteristic, as reported by others [94]. In addition, peaks appear in the all materials C1 ÷ C9, around 421 cm^−1^ to 508 cm^−1^, that correspond to a complex structure due to diversified compositions that contain aliphatic or small molecular groups from additives incorporates [95,96].

**Figure 16:**
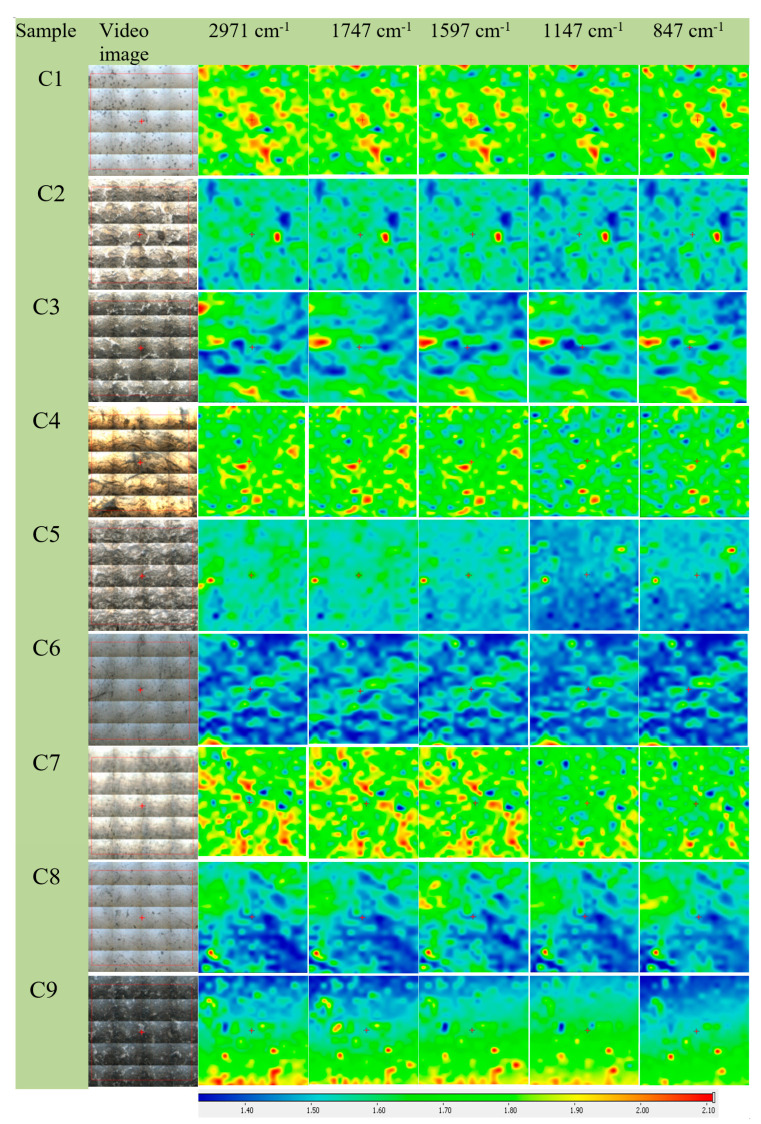
Presentation of video image and FTIR maps of experimental samples C1 ÷ C9; red areas indicate the highest absorbance, while blue areas correspond to the lowest absorbance.

To determine the spatial distribution for the antimicrobial additives (AgNPs) in the polymeric matrices C1 ÷ C9, FTIR microscopy analysis was conducted at 2971 cm^−1^, 1747 cm^−1^, 1597 cm^−1^, 1147 cm^−1^, and 847 cm^−1^. Figure 16 presents microscopic images with the delimitation of the analyzed areas together with maps corresponding to the mentioned wavenumbers.

Samples C1, C4, and C7 exhibit similar maps, expressing homogeneous compositions, depicted in an intense green color. Samples C2, C5, and C8 present compositional modifications shown by the green color change to light blue. Samples C3, C6, and C9 also present similar homogenous morphological characteristics, in dark blue, due to high AgNP concentration in the composition.

### 3.4. Thermal Analysis

Due to complex structures that are less crystalline but preponderantly syndiotactic [97,98,99], PVC allows for plasticization and compounding with numerous additives with the purpose of rigid or flexible composite preparation, with diverse applicability in the medical field industry, packaging, or other products. Generally, PVC exhibits thermal stability during melting, but the behavior and processing parameters depend on the chemical structure, physical properties, and content of the component substances.

**Figure 17:**
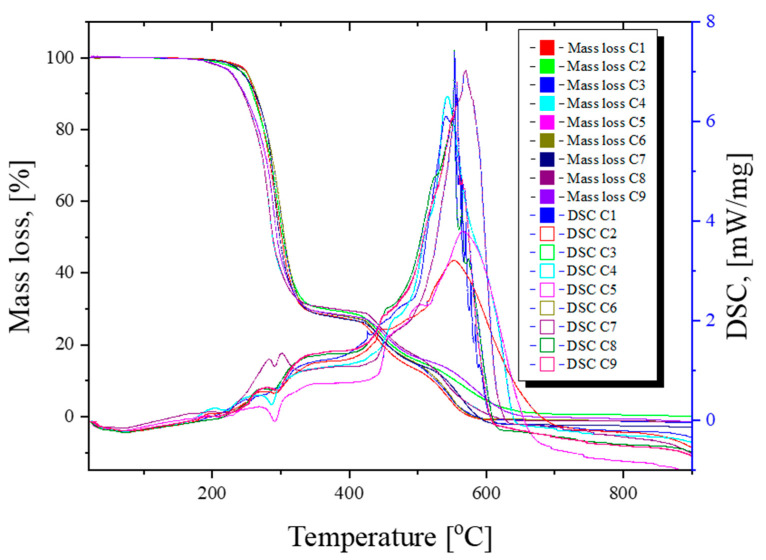
Graphic representation of DSC and TGA curves determined for plasticized PVC samples.

The results of the TG-DSC measurements, expressed as thermogravimetric curves, are presented in Figure 17.

According to the obtained experimental data, it can be observed that all samples are quite thermally stable, losing 1–3% from their initial mass up to 220 °C, the process being accompanied by very weak effects on the DSC curve around 70 °C. After this endothermic effect, which can be attributed to the elimination of residual solvent molecules or plasticizers, an exothermic effect after ~200 °C can be noticed due to partial oxidation.

The main degradation step takes place between 220 and 360 °C when the principal mass loss is recorded (~67–71%). The thermal effects on the DSC curve are complex, with small peaks, both endothermic and exothermic. This indicates that degradation is a complex process, comprising many superposed reactions, including both fragmentation of the polymer backbone and oxidation of the fragments. The residual carbonaceous mass is oxidized and burned away between 360 and 640 °C. The DSC curves present two important zones: around 470 °C, an exothermic effect, as a shoulder is observable due to partial oxidation of the polymer residuals, and a strong exothermic effect around 540–555 °C due to burning of carbonaceous mass. The principal numeric data are presented in Table 5.

The residue values obtained within the range of 360–640 °C exhibit a minimal decomposition of the studied materials, as well as good sample thermal stability.

### 3.5. Biological Properties

Numerous investigations concern both antimicrobial properties and cytotoxicity of AgNPs used as antimicrobial agents in different applications. Even though the mechanisms behind their action is mostly unknown or uncertain, it is clear that the chosen synthesis method, determining specific shapes of nanoparticles, influences the bactericidal/cytotoxic effect [100].

Here, as mentioned before, we used AC, which is a commercial antimicrobial agent comprising silver nanoparticles embedded in a ceramic matrix, and AP, i.e., silver nanoparticles prepared in our laboratories. Our results, as will be detailed further, show the difference between the two antimicrobial agents as well.

#### 3.5.1. Antimicrobial Analysis

**Figure 18:**
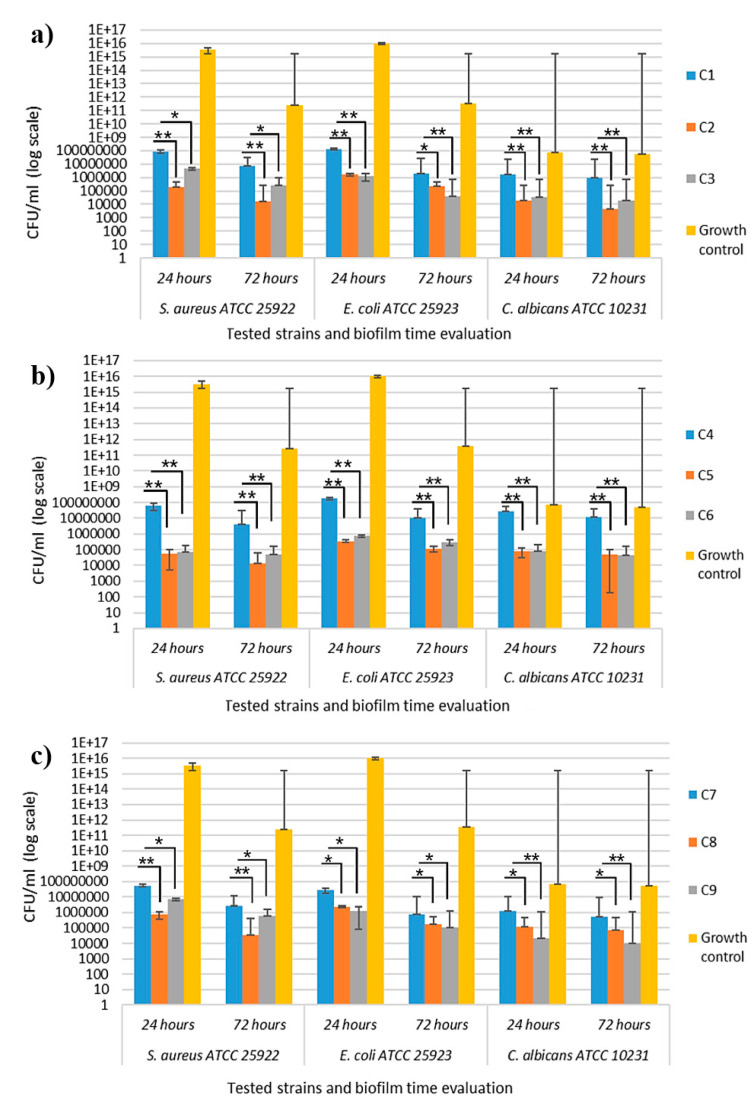
Graphic representation of CFU/mL values for quantitative evaluation of monospecific microbial biofilm growth after 24 and 72 h of incubation in static conditions: (**a**) C1 ÷ C3 samples, (**b**) C4 ÷ C6 samples, and (**c**) C7 ÷ C9 samples. Stars in the boxplots indicate the statistical significance (* for *p* < 0.05, ** for *p* < 0.001).

VCCs assessed from mechanical detachment of the microbial cells revealed a slightly different ability of the functionalized polymeric samples to inhibit biofilm generation and evolution. The quantitative results showed that sample C2, compared to the C1 control polymer, demonstrated anti-biofilm activity but only for Gram-positive *S. aureus* ATCC 25923 and *C. albicans* ATCC 10231 strains and sample C3, compared to the same control polymer, which demonstrated anti-biofilm activity for Gram-negative *E. coli* ATCC 25922 bacterial strain and *C. albicans* ATCC 10231 yeast strain (Figure 18a).

The same C2 samples inhibited the microbial cell adherence of for Gram-negative *E. coli* ATCC 25922 after 24 h of incubation, but the inhibitory effect was not maintained for a longer period, with the quantitative evaluation at 72 h showing a less significant inhibitory effect (Figure 18a). Samples C5 and C6, compared to the control polymer sample C4, significantly inhibited the microbial adherence (after 24 h of incubation) and biofilm maturation (after 72 h of incubation) of all tested strains (Figure 18b), demonstrating the best biological activities by preventing biofilm formation.

The inhibitory effect of samples C8 and C9 was manifested strictly dependent on the tested strains. Thus, sample C8 significantly inhibited the microbial adherence (after 24 h of incubation) and biofilm maturation (after 72 h of incubation) of the *S. aureus* ATCC 25923 bacterial strain, and sample C9 significantly inhibited microbial adherence and biofilm maturation in the *C. albicans* ATCC 10231 yeast strain (Figure 18c).

Overall, the best results were obtained for the composites incorporating AP, especially C6 and C9, even if antimicrobial agent concentration was lower than in the case of AC. The results can be correlated with the agglomeration tendency of the AP in polymeric compositions, as shown in SEM micrographs.

#### 3.5.2. Cytotoxicity Assessment

Cytotoxicity of materials when used in medical devices is always a concern. In our case, the toxicity can occur because of both plasticizers and AgNPs [100,101], as PVC is well known as being biocompatible [102]. Dose, aggregation, and particle sizes, are only some of the factors affecting a material’s ability to damage a living cell.

**Figure 19:**
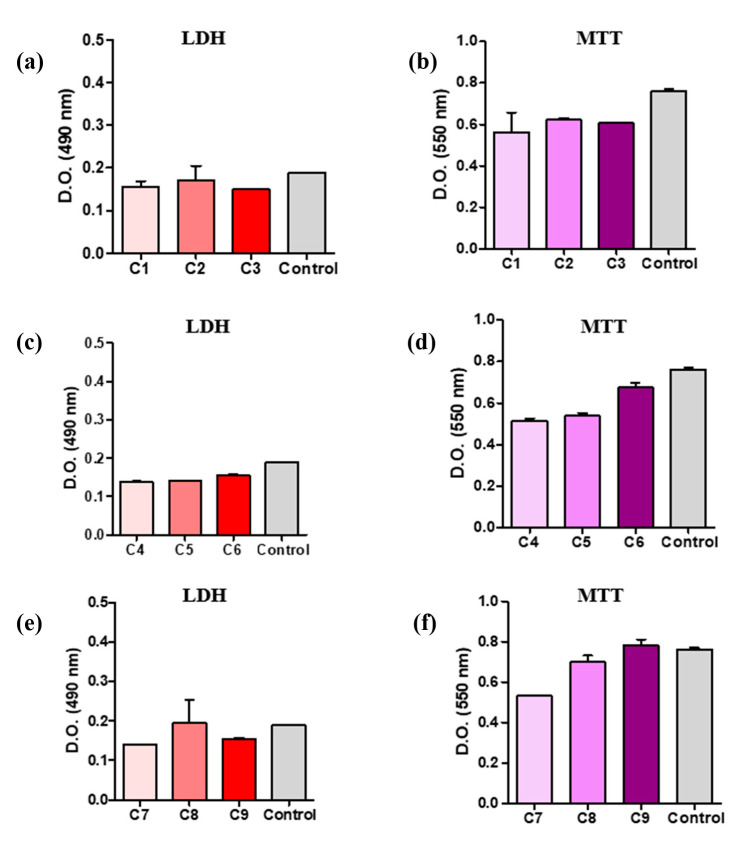
Graphic representation of values determined by LDL/MTT test: (**a**,**b**) C1 ÷ C3 samples, (**c**,**d**) C4 ÷ C6 samples, and (**e**,**f**) C7 ÷ C9 samples.

The results of Test Live/Dead are presented graphically in Figure 19. As can be seen, materials C1 ÷ C3 do not show cytotoxicity, but the level of cell proliferation is low compared to the control sample (cells under standard cultivation conditions). Materials C4 ÷ C6 do not show cytotoxicity, as shown by the LDH test. Among the three variants tested, the C6 material was associated with an increased degree of cell proliferation.

**Figure 20:**
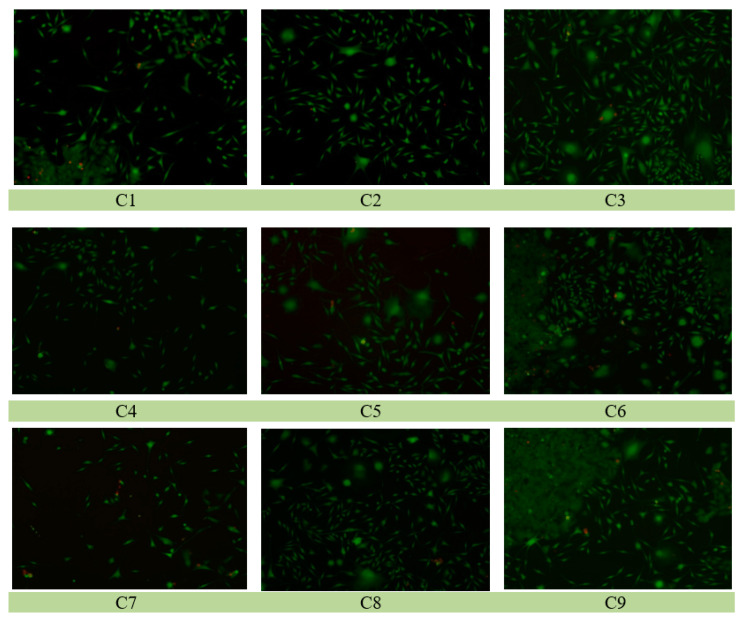
Microscopic images of cell cultures performed on surfaces of C1 ÷ C9 plasticized PVC samples.

Material C9 shows a high percentage of viable cells. In Figure 20, the graphs present viable cells that are shown in green color.

According to the above, the differences between samples are not significant, as all tested materials showed no cytotoxicity. However, the samples containing AP and P3 provide better environments in terms of cell proliferation. The results can be correlated with limited (almost zero) migration of plasticizers and the conditions of AgNPs.

Although the mechanisms are not well understood so far, the bactericidal and anti-biofilm actions of nanoparticles in general and of AgNPs in particular have intensively been researched and demonstrated [103,104,105]. The same observation is valid for water-based AgNPs suspensions, which may exhibit (lethal) cytotoxicity even at very low concentrations [102]. In the case of this study, some factors contribute to materials non-cytotoxic characteristics, despite their AgNP content. Thus, especially regarding AP, the most important can be mentioned: overall low AgNP concentration, rather large particle dimensions (average 30 nm), agglomeration tendency as proved by SEM micrographs, and immobilization (physical entrapment) within the polymeric matrix (and not surface deposition).

To conclude, as also observed by others, non-cytotoxic characteristics of the obtained materials are due to low AgNP concentration, NP immobilization, and polymeric matrix properties (surface charge and stability) [102,106,107].

## 4. Conclusions

Within this research, nine experimental compositions based on plasticized PVC with three types of plasticizers, DEHP (P1), Proviplast 2646 citrates (P2), and Proviplast 2755 biobased (P3), were developed. We aimed at the removal of the migration effect of the plasticizer by using two innovative and effective plasticizers, commercially available and accepted by the *European Pharmacopoeia*, like P2 and P3, as safer alternatives for P1 (commonly used nowadays). We also pursued the achievement of the antimicrobial effect of the polymeric compositions by using two types of additives based on AgNPs. The nine polymeric compositions were analyzed and physicomechanical, thermal, morphological, antimicrobial, and biocompatibility properties were evaluated. There were no significant differences found between the physicomechanical properties of C1 ÷ C9 samples. An increase in the hydrophilic character could be observed in the case of compositions added with AgNP powder, which would allow for at least a reduction in urethral trauma. At the same time, the use of AgNPs led to a slight but still acceptable increase in Shore A hardness. The migration of the plasticizer was lower when P2 plasticizer was used, in comparison with samples plasticized with P1 and P3. The tensile strength of C3, C6, and C9 samples was slightly higher than the other samples, as the antimicrobial additive AP acted as a filler in the mixture. At the same time, the elongation at break underwent changes corresponding to the type of antimicrobial additive. SEM microscopy micrographs revealed homogeneously mixed compositions, in which the antimicrobial additives were highlighted.

The antimicrobial compositions presented bactericidal activity against Gram-positive *S. aureus* ATCC 25923 and *C. albicans* ATCC 10231 strains and a Gram-negative *E. coli* ATCC 25922 bacterial strain. Among them C6 and C9 gave the best results.

So far, the results of this preliminary investigation are promising, as all studied parameters show compliance with quality standards for medicines and their ingredients. The obtained composite materials could be used for catheter development, to be further investigated and tested.

## Figures and Tables

**Table 1:** Compositions of experimental materials C1 ÷ C9, based on PVC.

Sample	PVC [g]	P1 [g]	P2 [g]	P3 [g]	ESO [g]	Ca/Zn Stearate [g]	Irganox 1076 [g]	AC [g]	AP [g]
C1	100	30	-	-	1.8	2	1.2	-	-
C2	100	30	-	-	1.8	2	1.2	2	-
C3	100	30	-	-	1.8	2	1.2	-	1
C4	100	-	30	-	1.8	2	1.2	-	-
C5	100	-	30	-	1.8	2	1.2	2	-
C6	100	-	30	-	1.8	2	1.2	-	1
C7	100	-	-	30	1.8	2	1.2	-	-
C8	100	-	-	30	1.8	2	1.2	2	-
C9	100	-	-	30	1.8	2	1.2	-	1

**Table 2:** Parameters applied for C1 ÷ C9 compounding.

Sample	Melting Temperature [°C]	Screw Speed [rpm]	Duration [min]
C1	170	75	10
C2	175	80	10
C3	176	78	10
C4	172	79	10
C5	175	81	10
C6	176	80	10
C7	173	75	10
C8	175	80	10
C9	176	78	10

**Table 3:** Pressing parameters.

Sample	Pressing Temperature [°C]	Preheating Time [min]	Pressing Time [min]	Cooling Time [min]	Total Cycle [min]	Pressure [bar]
C1	160	6	8	7	21	300
C2	165	6	8	7	21	300
C3	169	6	8	7	21	300
C4	173	6	8	7	21	300
C5	163	6	8	7	21	300
C6	167	6	8	7	21	300
C7	170	6	8	7	21	300
C8	174	6	8	7	21	300
C9	172	6	8	7	21	300

**Table 4:** Relative density, water absorption, and plasticizer migration, determined for experimental samples.

Sample	Relative Density [g/cm^3^] ISO 1183-1:2019 [55]	Water Absorption * [%] ISO 62:2008, Method 1 [54]	Plasticizer Migration ** [g] ISO 177:2016 [56]
C1	1.24	0.0005	0.008
C2	1.33	0.0004	0.0078
C3	1.35	0.0004	0.0069
C4	1.32	0.0004	0.005
C5	1.35	0.0003	0.0043
C6	1.34	0.0002	0.003
C7	1.33	0.0003	0.006
C8	1.35	0.0003	0.0059
C9	1.36	0.0002	0.004

* Maximal value admitted by European Pharmacopoeia 11 from 2023 is 0.0005. ** Maximal value admitted by European Pharmacopoeia 11 from 2023 is 0.02.

**Table 5:** DSC and TG parameters recorded for C1 ÷ C9 samples.

Sample	T_endo_ [°C]	Weight Temperature [°C]		Mass Loss [%] R_220 °C_	Mass Loss [%] R_220–360 °C_	Mass Loss [%] R_360–640 °C_
		T_5%_	T_10%_			
C1	73.4	253.2	261.8	1.04	70.43	29.52
C2	73.8	248.6	259.2	1.87	67.98	28.41
C3	73.5	253.6	264.1	1.22	69.74	29.89
C4	74.7	230.3	245.5	2.89	67.61	31.52
C5	70.8	231.9	246.8	3.10	67.85	28.37
C6	73.1	253.6	264.3	1.26	69.69	29.82
C7	70.3	247.2	262.8	1.30	70.35	30.50
C8	74.6	235.8	256.5	1.29	68.76	28.76
C9	74.9	241.7	259.3	1.27	67.89	29.81

## Data Availability

The original contributions presented in the study are included in the article, further inquiries can be directed to the corresponding author.
